# Predicting pharmacodynamic effects through early drug discovery with artificial intelligence-physiologically based pharmacokinetic (AI-PBPK) modelling

**DOI:** 10.3389/fphar.2024.1330855

**Published:** 2024-02-16

**Authors:** Keheng Wu, Xue Li, Zhou Zhou, Youni Zhao, Mei Su, Zhuo Cheng, Xinyi Wu, Zhijun Huang, Xiong Jin, Jingxi Li, Mengjun Zhang, Jack Liu, Bo Liu

**Affiliations:** ^1^ Yinghan Pharmaceutical Technology (Shanghai) Co., Ltd., Shanghai, China; ^2^ Jiangsu Carephar Pharmaceutical Co., Ltd., Nanjing, China; ^3^ School of Chemical Engineering and Pharmacy, Wuhan Institute of Technology, Wuhan, China

**Keywords:** artificial intelligence (AI), machine learning (ML), early drug discovery, PBPK modeling, PD modeling, P-CABs

## Abstract

A mechanism-based pharmacokinetic/pharmacodynamic (PK/PD) model links the concentration-time profile of a drug with its therapeutic effects based on the underlying biological or physiological processes. Clinical endpoints play a pivotal role in drug development. Despite the substantial time and effort invested in screening drugs for favourable pharmacokinetic (PK) properties, they may not consistently yield optimal clinical outcomes. Furthermore, in the virtual compound screening phase, researchers cannot observe clinical outcomes in humans directly. These uncertainties prolong the process of drug development. As incorporation of Artificial Intelligence (AI) into the physiologically based pharmacokinetic/pharmacodynamic (PBPK) model can assist in forecasting pharmacodynamic (PD) effects within the human body, we introduce a methodology for utilizing the AI-PBPK platform to predict the PK and PD outcomes of target compounds in the early drug discovery stage. In this integrated platform, machine learning is used to predict the parameters for the model, and the mechanism-based PD model is used to predict the PD outcome through the PK results. This platform enables researchers to align the PK profile of a drug with desired PD effects at the early drug discovery stage. Case studies are presented to assess and compare five potassium-competitive acid blocker (P-CAB) compounds, after calibration and verification using vonoprazan and revaprazan.

## Graphical Abstract

**Figure:**
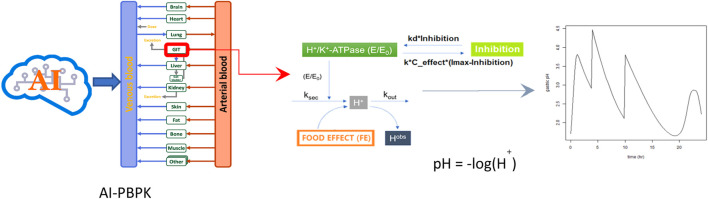
Main steps used to predict PK and PD outcomes of the compounds. (Step 1) Use different AI related simulations to predict the compound’s ADME and physiochemical properties. (Step 2) Predict PK outcomes using the PBPK model. (Step 3) PD models are used to predict how changes in drug concentrations affect gastric acid secretion and gastric pH. E/E0 is the relative activity of H^+^/K^+^ ATPase by drug; k_sec_ is the secretion rate constants for intra-gastric H^+^ concentration; k_out_ is the elimination rate constant for intra-gastric H^+^ concentration; H_obs_ is the observed concentration of H^+^; I (Inhibition) is the current antisecretory effect (or current pH level) of the drug; I_max_ is the maximum possible effect (or maximum pH level) of the drug can achieve; The term (I_max_ -I) represents how far the current effect is from its maximum potential.

## 1 Introduction

Drug discovery and development is a long and intricate process. It begins with identifying a biological target that can be affected by a drug to change disease progression. After validating this target, methods like virtual screening or high throughput screening (HTS) are used to screen through vast compound libraries, pinpointing ‘hits’ that show some activity. These hits are then refined into ‘lead’ compounds. These optimized leads are tested *in vitro* and on animals for safety and effectiveness checking. Essentially, drug development follows a process of broad to fine screening. We are now using the AI-PBPK platform to optimize the screening process from virtual compounds to candidate. The platform predicts ADME (Absorption, Distribution, Metabolism, Excretion) and physicochemical characters of the compound, and continuously predicts the human PK and PD outcomes of the drug candidate. Implementing this methodology in the discovery process could bring the clinical end points earlier and potentially lead to the identification of high-quality drug candidates earlier, ultimately reducing the timeline from target discovery to candidate selection.

PBPK modelling has become an essential component in the process of drug discovery and development. Within these models, established physiological characteristics and the ADME properties of a compound are incorporated into mathematical equations to predict drug behaviour in human body. The computational method involving model-based pharmacology evaluation was employed to determine the best range of PK, ADME and physicochemical characteristics for a specific target (Chen EP and Michalski, 2021). As a web-based platform, B^2^O simulator^®^ integrated PBPK models to predict drug exposure, potential interactions with other drugs and the likelihood of been bioequivalent with reference compound (Zhang et al., 2021a; Zhang et al., 2021b; Li et al., 2022; Li J et al., 2022; Zhang J et al., 2023). In those studies, drug related physicochemical parameters were searched online or calculated. Various computational techniques have been established for determining key parameters, including permeability (Paudel et al., 2023). When employing quantitative techniques to develop predictive models, the models could serve different purposes. For example, quantitative structure—toxicity relationship (QSTR) models were used for toxicity prediction based on chemical structure (Rai et al., 2023), whereas PK models were used to understand and predict the pharmacokinetic profile of drugs in the body.

**FIGURE 1:**
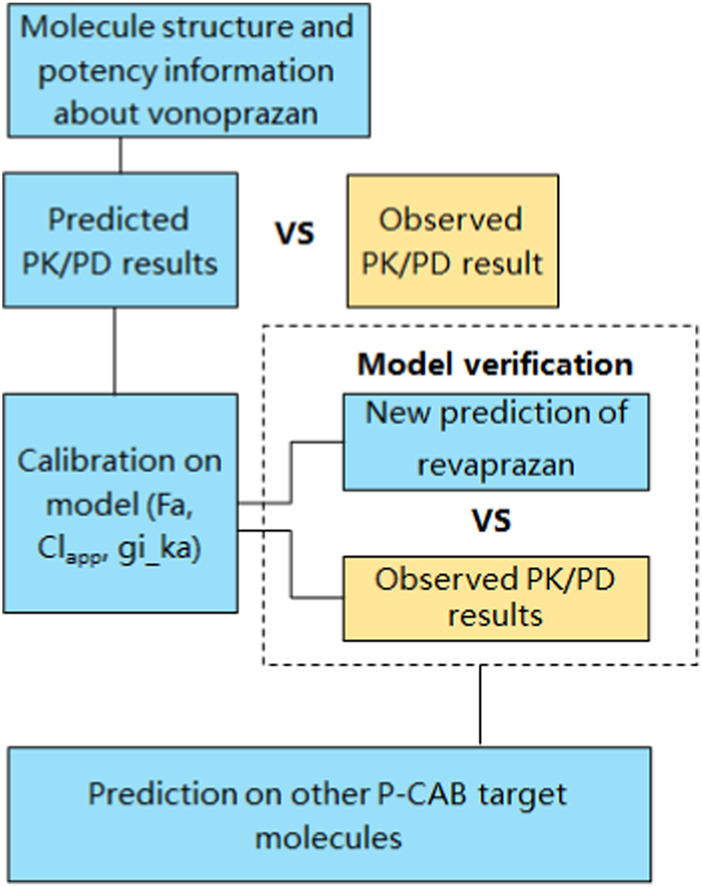
Workflow for predicting the PK and PD outcomes of a compound using AI-PBPK platform.

In the current study, the B^2^O simulator integrated the PD model, PBPK and AI related algorithms into one platform. Machine learning (ML) methods, as a subset of AI, are used to predict the ADME and other physiochemical properties. As a specific type of machine learning algorithm, Graph Neural Networks (GNNs) is used in this study to analyse chemical structures and to perform predication on structures’ PK parameters. Another algorithmic approach used in the study was random forest model. The platform employs the m5p random forest model, a decision tree-based regression method, for modelling parameters like apparent clearance (CLapp) (Alex A Freits and Ghafourian, 2015). Other global features such as logP, were calculated using the “CalcCripppenDescriptors” function in the RDKit cheminformatics toolkit. PKa was calculated using the open-source Dimorphit-DL (Ropp et al., 2019). The workflow for predicting the PK and PD outcomes of a compound using the AI-PBPK platform is shown in Figure 1. Examples such as potassium-competitive acid blocker (P-CAB) drugs were used to demonstrate the utility and benefits of this method in guiding early discovery studies. The process involved using the molecular structure and potency data of vonoprazan to calibrate its PK and PD outcomes against observed results. Following this calibration, the model underwent validation using revaprazan, another compound in the P-CABs class. Subsequently, the PK and PD outcomes for five different P-CABs were predicted and compared to identify the most promising candidate.

The PD model was adapted from a mechanism-based model developed by Kong et al., 2020 and Jia et al., 2021. It was indicated that a significant proportion (52%) of drug failure in clinical trials was due to poor efficacy from available data (Kiriiri et al., 2020). To prevent subsequent setbacks, it is crucial to identify and validate the target, confirming its significance in early discovery stage. “The proportion of time with a gastric pH > 4 over a 24-h period” is the endpoint in clinical studies of P-CAB drugs. P-CABs are designed to inhibit gastric acid secretion. The gastric pH level is a direct measure of acidity in the stomach. A pH greater than 4 indicates a reduction in gastric acidity. This endpoint can be predicted using mechanism-based pharmacokinetic (PK) and pharmacodynamic (PD) models. For P-CABs, PK modelling can predict the concentration of the drug in the bloodstream. It is helpful to understand the drug’s availability in the body, which is directly related to its efficacy. PD models are used to predict how changes in drug concentration affect gastric acid secretion and the gastric pH levels. The model considers the drug’s mechanism of action, such as how the P-CAB drugs inhibit acid secretion by blocking the H+/K + -ATPase (proton pump) in the stomach lining, which directly affects gastric acidity, and thus pH levels.

## 2 Methods

### 2.1 PBPK/PD modelling

The whole-body PBPK model, designed to predict plasma concentration of vonoprazan and other P-CABs PK profiles at different does, was based on two studies reported by (Thierry Wendling et al., 2015a; Thierry Wendling et al., 2015b). The model comprised 14 tissue compartments including lungs, hearts, brain, muscle, adipose, skin, spleen, pancreas, liver, stomach, gut, bones, kidneys and rest-of-body and 2 blood compartments (arterial and mixed venous). Each tissue compartment is assumed well-stirred, with the extent of distribution being characterised by the equilibrium tissue: blood partition coefficient (Kp). The rate equation for the tissue compartments can be expressed as follows (Eq. 1):

$$\frac{dA_{T}}{dt}=\frac{Q_{T}}{V_{VEN/ART}}∙A_{VEN/ART}-\frac{Q_{T}}{V_{T}K_{p}}∙A_{T}$$ (1)

Where A_T_ is the drug amount (μg), V_T_ is the volume (L) and Q_T_ is the blood flow (L/h) for the different tissues, and A_VEN/ART_ and V_VEN/ART_ are the amount (μg) and volume (L), respectively, of either mixed venous blood or arterial blood for all other tissues. Drug elimination is assumed to occur entirely in the liver compartment via oxidative metabolism (Thierry Wendling et al., 2015a). The PBPK model was integrated into the B^2^O platform. Subsequently, the PD model was also integrated and described the correlation between the plasma concentration of vonoprazan and the intra-gastric pH levels.

In the PD model, the antisecretory effects of the drug I, which also represented the increase pH, was calculated using the formula (Eq. 2):

$$\frac{dl}{dt}=k∙C_{e}∙\left(I_{\max}-I\right)-k_{d}∙I$$ (2)

where dI/dt is the rate of change of the drug’s antisecretory, which is also the rate of change of pH over time. I_max_ is the maximum possible antisecretory effect (or maximum pH level) the drug can achieve. K is the binding rate constant using the ration of k_d_ to k_i_. K_i_ is the inhibition index. C_e_ is the free drug concentration in the stomach and k_d_ is the irreversible inhibition efficacy of H^+^/K^+^—ATPase. E/E_0_ in Graphic abstract equalls to (1-I). C_e_ is calculated from the following formula (Eq. 3):

$$C_{e}=\frac{A_{GU}∙bpr}{K_{P_{gut}}∙k_{p_{scaler}}∙V_{GU}}∙fub$$ (3)

where A_GU_ is the amount of drug in gut and Volume of gut. K_p_ is the Tissue gut: plasma partition coefficient. Kp_scaler adjusted all K_p_ values in equal proportions. Bpr is the blood to plasma ratio and fub is unbound to plasma proteins in the bloodstream.

### 2.2 Graph neural networks

As a type of GNN model, The Message Passing Neural Network (MPNN) model was used following the work developed by (Gilmer et al., 2017; Jose Jimenez-Luna et al., 2021). The MPNN model operates on undirected graphs composed of vertexes (v) and edges (e) (Gilmer et al., 2017). It’s particularly useful for predicting molecular properties. The next two steps provide a simplified explanation of how the MPNN operates:

• Stage 1 Message Passing Phase: this phase runs for T time steps and involves two function-message functions (M_t_) and vertex update functions (U_t_). For each node in the graph, features from its neighbour vertexes and edges (e_vw_) are propagated into a message vector. The hidden states ( $h_{v}^{t}$ ) at each node are updated based on these messages. The equations are as follows (Eqs 4, 5):

$$m_{v}^{t+1}=\sum_{w\in N\left(v\right)}M_{t\;}\left(h_{v}^{t},h_{w}^{t},e_{\text{vw}}\right)$$ (4)

$$h_{v}^{t+1}=U_{t\;}\left(h_{v}^{t},m_{v}^{t+1}\right)$$ (5)

Here, w∈N(v) denotes the neighbour vertexes of each vertex v in the graph.

• Stage 2 Readout Phase: this phase computes a feature vector for the whole graph using a readout function (R). The readout function operates on the set of node states and must be invariant to permutations of the node states. The equation is as follows (Eq. 6):

$$ŷ=R\left(\left\{h_{v}^{t}\left|\;v\in G\right.\right\}\right)$$ (6)

These functions (M_t_, U_t_, R) are learned differentiable functions (Gilmer et al., 2017).

To train an MPNN model, one commonly employs a version of stochastic gradient descent. During this process, the model is trained to identify underlying structural traits of molecules and to predict molecular characteristics by minimizing the difference between its predictions and the actual values. This study highlights the application of MPNN models in clarifying the relationship between molecular structures and their properties, with a reference to the studies conducted by Jose Jimenez-Luna et al., 2021. In this example, all molecular structures were converted into graph representations using the Python DGL package. Additionally, 2D features produced by RDkit were integrated, similar approach was also used in random forest model. Parameters such as plasma protein binding rate (ppbr, data size: 4637, best fold *R* ^2^ = 0.551), blood to plasma ratio (bpr, data size: 461; best fold *R* ^2^ = 0.107), Volume of distribution (V_ss_, data size: 1305, best fold *R* ^2^ = 0.413) and Caco-2 Permeability (Papp, data size: 525, best fold *R* ^2^ = 0.412) were predicted. Permeability in Caco-2 cells was used to calculate Fa, the fraction absorbed. The model of the best fold was chosen for the AI-PBPK model.

### 2.3 Random forest (tree model) models

The m5p random forest model consists of an ensemble of m5p trees, which are decision trees that use linear regression functions at the leaf nodes instead of constant values. The m5p random forest model works by randomly selecting a subset of features and a subset of instances for each tree, and then building the tree using the m5p algorithm. The m5p algorithm splits the instances based on the feature that minimizes the variance of the linear regression functions at the child nodes. The final prediction of the m5p random forest model is obtained averaging the predictions of all the trees in the ensemble. More details of how the algorithm trains a model and performs prediction were addressed in Freitas’s original work (Alex A Freits and Ghafourian, 2015). After design and coding, data was trained for Clapp model. The Clapp dataset was split into 10 folds, each were successively deemed as validation set in each turn and the rest 9 folds were deemed as a training set. The mean absolute error (MAE) was chosen as the indicator to show the performance of the model trained by the training set of each turn. The smallest MAE of all turns described how well the model fits the training data and predicts external data and helped to choose the best Clapp model to be added to the AI-PBPK model. Parameters such as apparent clearance CL_app_ (data size: 98, best fold *R* ^2^ = 0.388, internal *R* ^2^ = 0.7996) was predicted using this model.

### 2.4 Data source of machine learning

Five ADME parameters were modelled by machine learning. They are the fraction of the drug that is unbound to plasma proteins in the bloodstream (fup; fup = 1-ppbr), steady state volume of distribution vss_perKg, apparent clearance Clapp (Clapp = vss*0.693/half-life), gastrointestinal absorption constant (gi_ka; gi_ka = 2*Papp/radius of small intestine) and blood to plasma ratio (bpr). The radius of small intestine is 1.25 cm according to its 2.5 cm diameter (Herbert and Helander, 2014). The data for the first 4 parameters were collected from the Therapeutics Data Commons (TDC) database. Its Python package named “PYTDC” was installed, and the correspondent datasets were used in this package:

**Table:** 

Datasets	ADME parameters	Short names
TDC.PPBR_AZ	Plasma protein binding ratio	Ppbr
TDC.VDss_Lombardo	Steady state volume of distribution	Vss
TDC.Half_Life_Obach	Half life	
TDC.Caco2_Wang	Caco2 permeability	Papp
Mamada’s work (Hideaki Mamada et al., 2021)	Blood to plasma ratio	Bpr

Additionally, the bpr data were from the Mamada’s work (Hideaki Mamada et al., 2021). All 5 datasets were composed of 2 vectors with same lengths. One of them was their molecular structure displayed in SMAILES codes, and the other was the correspondent ADME parameter values for each molecule.

### 2.5 Design of simulation studies

When the PBPK/PD model was established, and the needed parameters were collected or generated by machine learning models, the PK and gastric pH profiles were simulated for each drug for a 48—hour duration after 20 mg single oral dose.

### 2.6 Case study: potassium-competitive acid blocker drugs to reduce the production of stomach acid

As one of the most well-known examples of P-CAB drugs, vonoprazan has gained global recognition to treat conditions related to stomach acid, such as gastroesophageal reflux disease (GERD) and peptic ulcers. Its primary target is the H^+^/K^+^ ATPase enzyme. Known as the proton pump, the enzyme is located in the stomach lining. Vonoprazan inhibits the proton pump by binding competitively to the potassium-binding site on the pump, thus inhibiting the exchange of hydrogen ions for potassium ions. This inhibition reduces the amount of gastric acid produced and secreted into the stomach, which can help to alleviate symptoms associated with acid-related disorders. In clinical trials, the proportion of time with a gastric pH > 4 over a 24-h period is often used as a primary or secondary endpoint. Because the optimal PK required for a drug candidate to elicit efficacy is highly dependent on the targeted pharmacology (Chen EP and Michalski, 2021), PK and PD results were both analysed in the platform to avoid misguiding of compound screening and design. The study also included five other compounds which also has H^+^/K^+^ ATPase as the target.

In this study, the PK performance of vonoprazan was firstly predicted and calibrated with observations. After verification with revaprazan, the PK performance of the other five P-CAB compounds was simulated. Following that, their PD outcomes were also simulated using the AI-PBPK platform. Finally, the PK and PD results of different doses of compounds were analysed and compared, and the optimal combination of PK, PD and dose of candidate compounds was determined.

### 2.7 Literature search

Observed data were collected from literature search. Since vonoprazan was firstly approved in Japan in 2014, a PubMed search was conducted using “vonoprazan” AND “clinical trial” as keywords, beginning from the year 2014. Only those publications that contain comprehensive PK data from a 20 mg monotherapy of vonoprazan in healthy subjects for the first 2 days were included. Observed PD data were collected from literature search as well, with the same keywords. We only included studies that provided results on gastric pH *versus* time over a 24-h period from a 20 mg monotherapy of vonoprazan in healthy subjects (fasting condition).

### 2.8 Software

The PBPK model was implemented using the software B^2^O simulator, a web-based platform, to predict drug exposures. With lower and upper CI% (Confidence Interval) limits 2.5%–97.5%, the geometric mean of the maximum serum concentration that a drug achieves in the body (C_max_) and the area under the curve (AUC) were calculated, and ratios between geometric means were calculated and compared with the observations from clinical studies. Changes bigger than or equal to 2-fold were considered significant.

### 2.9 Statistical analyses

Pearson’s correlation coefficient (r) is a statistical measure that express the extent of the correlation between two variables. The correlation coefficient was calculated using R version 4.3.0.

Local sensitivities of the following 17 parameters predicted by the machine learning models were analysed. They were fup, bpr, gi_ka, clapp, vss_perKg, kp_bone, kp_brain, kp_adipose, kp_heart, kp_kidney, kp_gut, kp_liver, kp_lung, kp_muscle, kp_skin, kp_spleen, kp_scaler. Kp represents the tissue: plasma partition coefficient. Each of them was tuned ±1% from their predicted value, and the percent change of AUC and Cmax values of revaprazan in response to an average 1% change in each parameter were studied and compared.

## 3 Results

### 3.1 Simulation, calibration and verification of PK behaviour

#### 3.1.1 Simulation of vonoprazan PK performance

**FIGURE 2:**
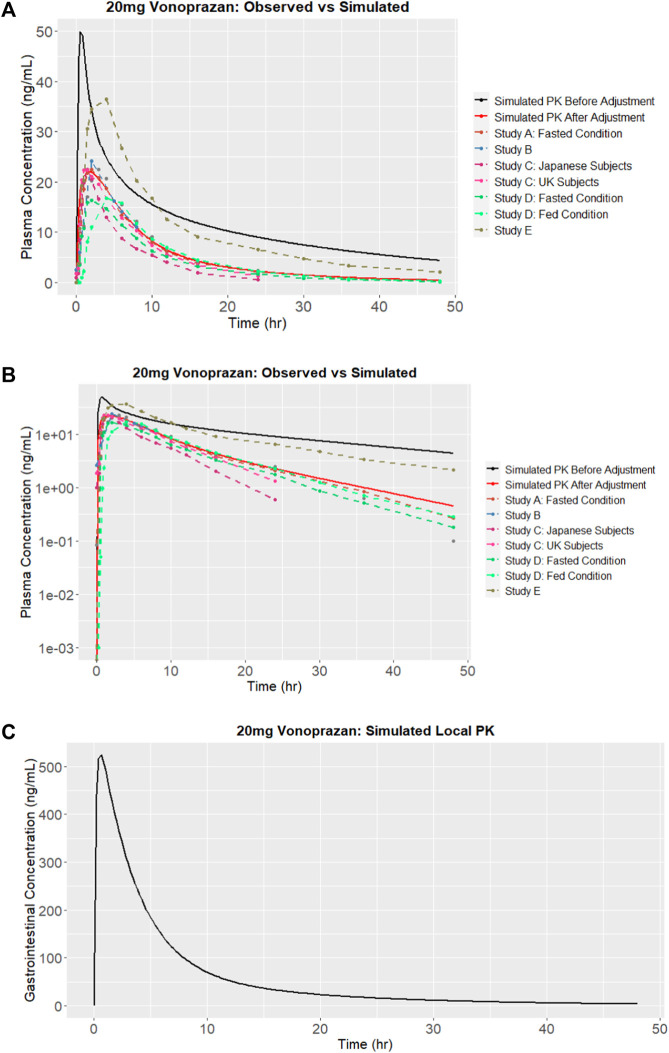
Simulation results of vonoprazan: **(A)** Plasma concentration *versus* time and comparisons with observations from studies A (Kentaro, 2018), B (Tack et al., 2023), C (Jenkins et al., 2015), D (Mulford DJ et al., 2022) and E (Jenkins and Patat, 2017), before and after calibration (adjustment); **(B)** Logarithm of simulated vonoprazan plasma concentration *versus* time before and after calibration (adjustment); **(C)** Concentration in the stomach after calibration.

The SMILES code for vonoprazan is CNCC1 = CN(C (=C1)C2 = CC = CC = C2F)S (=O)(=O)C3 = CN = CC = C3. When the SMILES code was entered into the AI-PBPK platform, the plasma concentration of vonoprazan was simulated and the result is shown in Figure 2A (solid black line). The predicted parameters including ADME and physicochemical properties, are listed in Table 1. The Kp scaler adjusted all Kp values in equal proportions. Its size varied based on the predicted vss_per Kg. The outcomes from the simulation were compared with the observed results. After literature search, PK data observed from five clinical studies were chosen, and their values were used for comparison with the simulation outcomes, as described in Figure 2A. From the figure we can see that the simulated drug exposure, which is represented by the area under the curve in the plasma concentration *versus* time plot, was greater than the values corresponding to the average observed exposure.

**TABLE 1:** Predicted parameters for vonoprazan.

Fraction unbound in plasma, fup	0.13418	Apparent elimination rate, Clapp	11.58736
Blood-to-plasma ratio, bpr	0.80581	Volume of distribution, vss_per Kg (L/kg)	3.497
Absorption rate, gi_ka	1.51647		
Tissue: plasma partition coefficient K_p__			
Bone	3.28785	Liver	13.84071
Brain	90.15343	Lung	6.48797
Adipose	29.91066	Muscle	7.44037
Heart	13.85107	Skin	56.12225
Kidney	20.40353	Spleen	7.81823
Gut	14.27052	K_p_ Scaler	0.3012

#### 3.1.2 Calibration with vonoprazan PK performance

In this case, the terminal half-life, which represents the elimination of drug, should be adjusted. In order to determine the extent of reduction needed, the log of plasma concentration *versus* time for both simulated and observed results are plotted in Figure 2B. Since the slope of the logarithmic curve represents the terminal half-life and the slope of the predicted log curve was about 3.08 times that of the observed curve, the initial simulation leads to a much quicker absorption. In this case, the drug absorption fraction Fa, was adjusted to be half of its original predicted value. Simultaneously, the elimination CL_app_ was adjusted 3.08 times bigger and the absorption rate (gi_ka) was adjusted 3.5 times smaller. The PK outcomes before and after adjustment are listed in Table 2, and the predicted results following parameter adjustment are also shown in Figure 2A.

**TABLE 2:** Comparison of simulated PK results before and after adjustment.

PK Parameters	Simulated results before adjustment	Observation Kentaro, (2018)	Simulated results after adjustment
AUC_0-t_ (ng*hr/mL)	1138.4	223.9	241.14
AUC_0-∞_ (ng*hr/mL)	1435.2	227.1	248.14
C_max_ (ng/mL)	99.7	22.5	22.01
T_max_ (hr)	0.6	2	1.8
Terminal T_1/2_ (hr)	23.48	7.62	10.68

AUC: the area under the curve.

C_max_: the maximum serum concentration that a drug achieves in the body.

T_max_: time to peak drug concentration.

T_1/2_: the time required for half the dose of drug administered to be removed from the body.

#### 3.1.3 Mode verification with revaprazan

**FIGURE 3:**
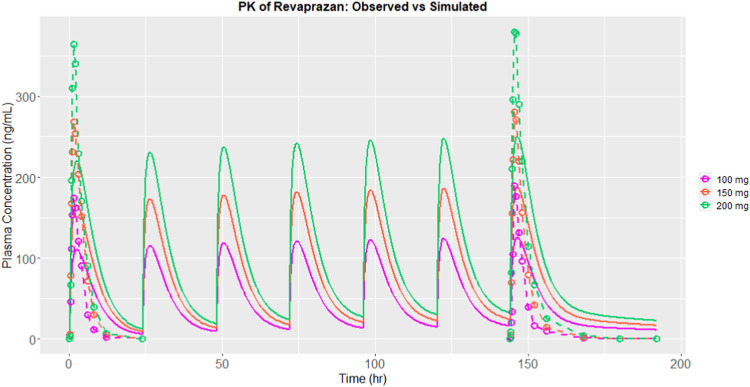
Simulated (solid lines with colours) and observed plasma concentration (dashed lines with colures) of revaprazan after calibration.

After calibration, the predictive capacity of the platform was further validated by inputting the chemical structure of revaprazan, which also has P-CAB target. The predicted PK outcomes were compared with observed results (Kim et al., 2010), as described in Figure 3. Three different doses (100 mg, 150 mg, and 200 mg) were used and the results on days 1 and 7 were compared. The predicted and observed data of revaprazan after orally taken 200 mg on days 1 and 7 are listed in Table 3. All the outcomes were simulated based on one virtual healthy subject. From the table we can see that the predicted PK results are within two times of the observed results. The Pearson’s correlation coefficient (r) was calculated for all time points of the predicted and observed PK data of revaprazan at doses of 100 mg, 150 mg and 200 mg. As a result, the correlation coefficient was 0.8404, 0.8862, and 0.8754 respectively. The analysis results indicated that the model reasonably predicted drug exposure. In the early stages of drug discovery, using a model based on one single virtual healthy subject can provide a baseline understanding of how a typical healthy body might respond to the drug, which is useful for initial screening. Moreover, at this early stage, accurately defining the disease population remains challenging. The presence of disease could introduce considerable inter-variability of patients into the PBPK models, making it difficult to model multiple subjects in the early stage of drug discovery.

**TABLE 3:** Predicted and observed data of revaprazan after oral administration 200 mg.

Days		Predictions			Observations		
		AUC_0-t_ (ng*hr/mL)	C_max_ (ng/mL)	T_max_ (hr)	AUC_0-t_ (ng*hr/mL)	C_max_ (ng/mL)	T_max_ (hr)
1	Geometric mean	2091.2	221.2	2.5	1452.3	402.2	1.7
7	Geometric mean	2700.5	250.8	2.5	1916.1	453.6	1.7

AUC: the area under the curve.

C_max_: the maximum serum concentration that a drug achieves in the body.

T_max_: time to peak drug concentration.

Because it is believed that the concentration of vonoprazan in the stomach is different from the plasma concentration and could be 1000 times higher than the concentration in plasma 24 h after administration to humans (Kong et al., 2020), we simultaneously predicted the concentration of vonoprazan in the stomach, as shown in Figure 2C. Comparing to the plasma concentration, the peak concentration in the stomach was around 524 ng/mL, which was 23.8 times higher than plasma concentration in Figure 2A.

#### 3.1.4 Sensitivity analysis

The results of sensitivity analysis demonstrated that the most sensitive parameter that influenced the AUC and Cmax of PK prediction was bpr, which caused almost 1.5% change in Cmax and 1% change in AUC when it was changed by 1%. Moreover, CLapp caused approximately 1% opposite change in AUC when it was changed by 1%. The remaining parameters showed minimal sensitivity to PK predictions, as a local 1% alteration in their values results in less than 0.5% change, indicating they did not significantly influence the prediction.

### 3.2 Simulation, calibration and verification of PD outcomes

#### 3.2.1 Simulation and calibration of vonoprazan PD outcomes

When assessing acid-suppressing drug like P-CABs, maintaining a gastric pH greater than 4 for as much of a 24-h period as possible is often a critical PD endpoint. This is because a gastric pH above 4 is generally considered as the necessary threshold to prevent symptoms associated with acid-related disorders. To assess the PD results of vonoprazan, potency related parameters like IC_50_ (half maximal inhibitory concentration), K_i_ (the inhibition constant), and other pharmacological parameters such as Dissociation half-life (residence time), Kd (dissociation constant) and K (binding rate constant for the reaction between the drug and its target, K_d_/K_i_) were searched and calculated from literatures and the values are listed in Table 4. Vonoprazan and KFP_H008 showed relatively low IC50 values as 0.019 and 0.029 μM respectively, indicating higher potency of the compounds to inhibit gastric acid secretion. Revaprazan had the highest IC50 value as 1 μM. The time required for half the dose of drug administered to be removed from the body (t_1/2_) of dissociation of TAK-438 binding was 4.7 h. This value was employed for other P-CAB compounds because there was no available data from literature search. K_d_ was calculated from (ln (2)/dissociation half-life) and K was the binding rate constant using the ration of K_d_ to K_i_

**TABLE 4:** Input parameters for the prediction of PD outcomes.

Name	SMILES code	IC_50_ (μM, pH = 6.5)	K_i_ (μM)	Dissociation half-life (hr)	K_d_ (hr^-1^)	K (μM^-1^*hr^-1^)
Vonoprazan	CNCc1cc (-c2ccccc2F)n (S (=O)(=O)c2cccnc2)c1	0.019 Yasunobu Hori et al. (2010)	0.0095	4.7 Jai Moo Shin et al. (2011)	0.147	15.524
Revaprazan	CC1C2 = CC = CC = C2CCN1C3 = NC(=NC(=C3C)C)NC4 = CC = C(C=C4)F	1 Jai Moo Shin et al. (2011)	0.5	4.7	0.147	0.294
Compound −1	CCNCc1cc (-c2cccs2)n (S (=O)(=O)c2ccc(C)cc2)c1	0.31 Mitsuyo Kondo et al. (2012)	0.155	4.7	0.147	0.951
Compound −2	CCNCc1cc (-c2ccc3c (c2)OCO3)n (S (=O)(=O)c2ccc(C)cc2)c1	0.54 Mitsuyo Kondo et al. (2012)	0.27	4.7	0.147	0.546
KFP_H008	CNCc1cc (-c2ccc3 [nH]ccc3c2)n (S (=O)(=O)c2cccnc2)c1	0.029 Li et al. (2017)	0.0145	4.7	0.147	10.171
AZD0865/Linaprazan	Cc1cccc(C)c1CNc1cc (C(O) = NCCO)cn2c(C)c(C)nc12	*0.13 (Luo and Zou. (2014)	0.065	4.7	0.147	2.269
SCH28080	Cc1nc2c (OCc3ccccc3)cccn2c1CC#N	0.17 Mitsuyo Kondo et al. (2012)	0.085	4.7	0.147	1.735

*IC_50_ was measured under pH = 6.4.

**FIGURE 4:**
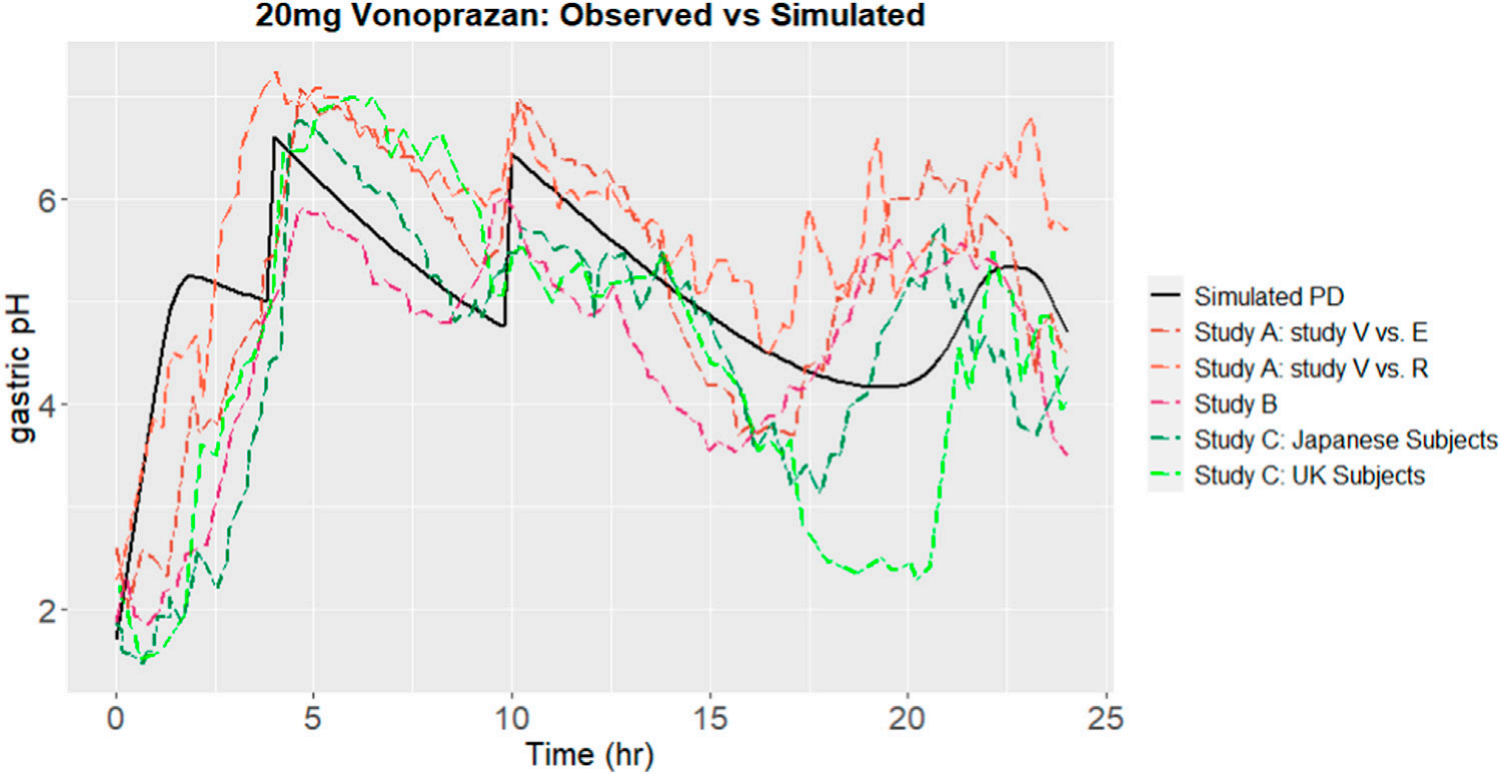
Simulated gastric pH over 24 h, comparing to observations from Studies A (Sakurai Y et al., 2015), B (Laine et al., 2022) and C (Suzuki et al., 2018), after calibration.

Using parameters sourced from literatures, the gastric pH of subjects after oral administration of vonoprazan was simulated. The outcomes from the simulation were compared with the observed results. Gastric pH is affected by the local concentration of proton pumps. Proton pumps, which is also known as H^+^/K^+^ ATPase enzymes, are present in the parietal cells of the stomach lining. Since proton pumps are responsible for the production of stomach acid, drugs like P-CABs are taken orally to inhibit these pumps, thereby reducing the secretion of stomach acid. The gastrointestinal concentration of drug doesn’t equal to the local concentration of the proton pump. As such, an enrichment coefficient for the drug, with a value of intersys = 1 × 10^3^, was introduced to the free vonoprazan concentration C_e_ in the stomach. After calibration, the predicted outcomes, together with observed results are shown in Figure 4. The predicted time percentage with a pH > 4 for vonoprazan was 88.88%. This was within a 1.5-fold variance compared to the mean of the clinical observations A-C (Sakurai Y et al., 2015; Suzuki et al., 2018; Laine et al., 2022), which averaged at 76.4%.

#### 3.2.2 Model verification with revaprazan

The ability of the platform to predict PD results was further verified by revaprazan with potency and relevant parameters. The predicted gastric pHs were compared with observed results. When revaprazan was administered orally at a dose of 200 mg, the predicted duration with a pH > 4 was 57.1%. This was within a 1.5-fold variance from the study observation, which recorded a value of 42.2% (Kim et al., 2010). The predicted onset time to reach pH 4.5 was 4 h. The observed time for intragastric pH to reach 4.5 was withing 2 h (Kim et al., 2010). All the predicted results indicate a reliable predictive capacity of the platform for revaprazan.

### 3.3 Prediction of PK and PD outcomes of other P-CAB compounds under the same dose

The PK and PD outcomes of the other five compounds were simulated under the same dose of 20 mg, which is the same to vonoprazan. Having P-CAB as the target, all the chemical structures were searched from literatures. Their names and SMILES codes are listed in Table 4.

**FIGURE 5:**
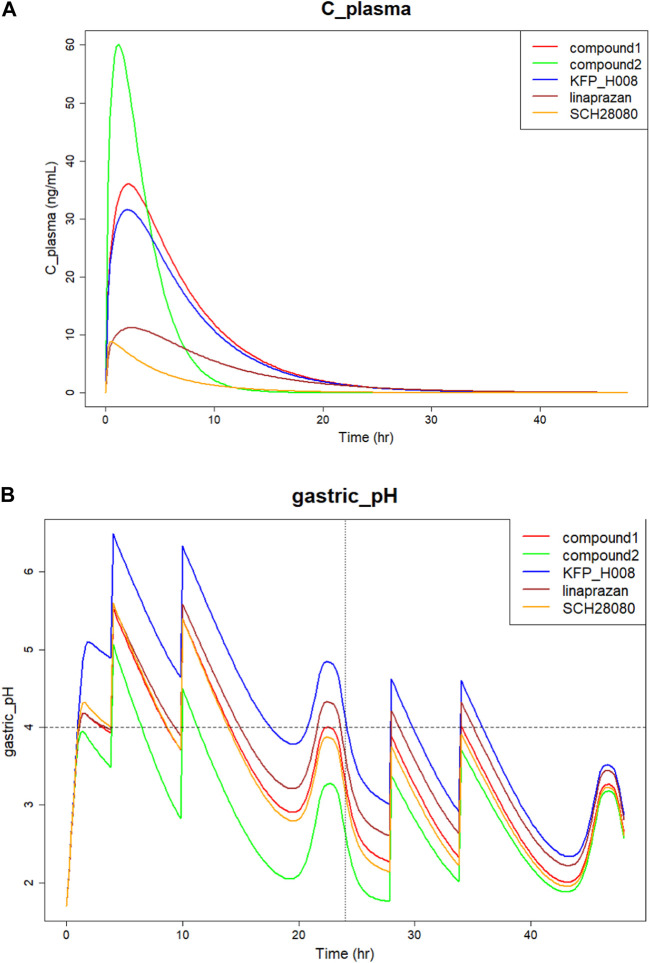
Simulation of five P-CAB compounds of **(A)** Plasma concentration *versus* time; **(B)** Gastric pH over 24 h after orally administration.

The comparison of predicted plasma concentration is shown in Figure 5A. From the results we can see that the five compounds showed similar plasma profiles with compound-2 having the highest concentration in around 2 hours, followed by compound-1. The simulated gastric pH *versus* time was compared and showed in Figure 5B. As mentioned above, pH > 4 holding time percentage is an important clinical indicator of P-CAB drug in the clinical development of Gastroesophageal Reflux Disease (GERD) and peptic ulcer and pH > 4 helps avoid irritation of the esophagus or ulcer by stomach acid. The shorter the onset of action, the faster the effect of the drug in relieving symptoms. From the figure we can see that within 24 h, all compounds showed fluctuating gastric pH around pH 4. A similar pH fluctuation was observed following the oral administration of vonoprazan, although vonoprazan exhibited a higher gastric pH value. The results of the 24-h time percentage and onset time for pH > 4 are summarized in Table 5. Combining the two major indicators, the simulation found that among the five compounds, KFP_H008 had the best percentage pH > 4 holding time with relatively short onset time, which were 81.17% and 0.99 h respectively. When compared to vonoprazan, KFP_H008 demonstrated close PD outcomes in terms of pH > 4 hold time and time of onset and showed promise for further development.

**TABLE 5:** Simulated pH > 4 holding time percentage and time of onset of P-CAB compounds under dose of 20 mg.

Drugs	pH > 4 Holding Time Percentage %	Time of Onset (h)
Vonoprazan	88.88	1.028
Compound-1	47.79	1.144
Compound-2	21.88	1.272
KFP_H008	81.17	0.99
Linaprazan	56.79	1.078
SCH28080	47.87	1.05

### 3.4 Prediction of PK and PD outcomes of other P-CAB compounds at different doses

The PD efficacy of different compounds at different doses were simulated to compare the development potential of the compounds. We compared gastric pH changes for different compounds at 10 mg, 20 mg, 40 mg, 80 mg, 160 mg, and 400 mg. With an increase in the dose from 10 to 400 mg, the pH > 4 holding time of most compounds increased. Starting with a dose of 20 mg, the percentage holding time of the KFP_H008 fluctuated from 80% to 90%. The results of pH > 4 holding time and the time of onset are summarised in Tables 6 and 7. Comparing to other compounds, KFP_H008 maintained the pH > 4 for the longest duration and had a comparatively faster onset time among all the doses, starting from 10 mg.

**TABLE 6:** PH > 4 holding time percentage % of different compounds under different doses.

pH > 4 Holding Time Percentage %						
Doses (mg) Drugs	10	20	40	80	160	400
Compound-1	36.68	47.79	95.51	70.60	79.71	88.53
Compound-2	15.77	21.88	28.07	35.05	41.95	49.25
KFP_H008	71.96	81.17	88.21	92.31	94.60	95.50
Linaprazan	43.54	56.79	69.23	79.63	87.60	93.15
SCH28080	37.24	47.87	57.82	67.42	76.48	86.69

**TABLE 7:** The onset time of different compounds under different doses.

Time of Onset (h)						
Doses (mg) Drugs	10	20	40	80	160	400
Compound-1	0.99	1.144	1.062	1.038	1.006	1.012
Compound-2	0.974	1.272	1.094	1.03	1.012	1.016
KFP_H008	1.024	0.99	1.036	1.002	0.996	0.994
Linaprazan	0.994	1.078	1.024	1.016	1.038	0.974
SCH28080	0.998	1.05	1.018	1.026	0.988	1.01

## 4 Discussion

It is believed that predicting PD, such as pH over time, using the AI-PBPK/PD model is more effective than predicting PK to accelerate the screening of compounds in the drug development stage. Since the primary interest in drug development is the therapeutic effect of drug, predicting the PD allows for a direct understanding of whether a compound is likely to have the desired therapeutic effect or not, and can streamline the compound screening process.

In the current study, ML algorithms are used to estimate model parameter. RDKit package in Pthyon is a cheminformatics library and it is specially designed for the analysis of chemical data. It can read various chemical file formats and represent molecular structures programmatically. And the RDKit compute a wide range of molecular descriptors and features such as molecular weight, log P, and hydrogen bond donors/acceptors. In DGL package, molecules can be represented as graphs, where atoms are nodes, and bonds are edges. It is powerful in learning the patterns and relationships in graph data. Coded and defined by DGL, GNNs can learn complex relationships between atom in molecule, which can be crucial for understanding tis properties and behaviour. Using DGL, the features extracted by RDKit can be integrated into the graph model. This allows the GNN to consider detailed chemical information while making predictions. This combination allows for a more holistic analysis of chemical compounds, considering both of their structural features and the complex interatomic relationships. High-quality, accurate, and well-annotated data is essential for training reliable predictive models.

Using ML or AI in drug development process is an important part of pharmaceutical companies’ strategies. Comparing to totally new drug development, “fast follow” study is based on a substantial data available from the pioneering drug, including its structure, model of action, therapeutic target, and clinical trial results. These data can provide a solid foundation for training machine learning models, potentially leading to more accurate predictions for new compounds. However, integrating AI with PBPK models presents numerous challenges and comes with its own set of limitations. Firstly, ML or AI models often require large datasets to be effective. In the current study, the majority of the parameters were predicted using data size exceeding 461, with exception of the apparent elimination rate CL_app_, which was estimated using a data size of 98. Increasing the data input is necessary to enhance the accuracy of the model’s predictions of parameters. Also, the training database has not included first-in-class structures. A first-in-class medication is a pharmaceutical that uses a “new and unique mechanism of action” to treat a particular medical condition. Secondly, any improvements to the PBPK model’s predictive accuracy brought about by AI need to be rigorously validated using experimental data. Thirdly, a single virtual subject cannot represent the variability and diversity found in a real population. Upon comparing with the observed data, both the PK and PD of the model demonstrated a close match. This similarity was further evidenced by a relatively strong correlation with the observed data, as demonstrated by Pearson correlation coefficients (r values) around 0.8. This indicates that the model performed well. In the present research, the model was calibrated by using the observed data from vonoprazan. After the calibration, another external validation was used to further validate the model by testing it against data sets that were not used in the calibration. This process ensures that the model is accurate and reliable in predicting real-world outcomes.

Sharing with similar core structures, P-CABs reduce stomach acid production within a few hours of intake, and often has a long duration of action. One well-known P-CABs is vonoprazan. It has been studied and used in various countries, offering an alternative to Proton pump inhibition (PPIs), especially in cases where PPIs are not effective or suitable. While like all medications, vonoprazan can have side effects. Common side effects include diarrhoea, constipation, abdominal pain and nausea. Also, drug interactions with other medications also need to be considered carefully. Revaprazan didn't demonstrate sufficient efficacy and failed to go onto market. In pharmaceutical industry, the drugs that are chemically similar to already existing drugs are called “me too” drugs. The strategy of developing “me too” drugs is adopted by companies aiming to enter a profitable market by creating a product that is similar to a top-selling drug. In this case study, the AI-PBPK platform becomes an effective and efficient way to find out the alternative “me too” drugs to vonoprazan. The possible PD outcomes of five P-CAB compounds were simulated using the same AI-PBPK platform after verification. KFP_H008 showed a comparatively longer duration with a pH > 4 compared to the other compounds, with a percentage exceeding 80% when the dose was more than 20 mg.

IC50 and Ki are both crucial parameter in biochemistry and pharmacology, and they help in understanding the type and strength of the inhibition. For IC50, a lower value indicates higher potency, meaning less substance is needed to achieve a 50% inhibition. In Table 4 with parameter values, vonoprazan showed the lowest IC50 and Ki values, followed by KFP_H008. Comparing to the other four compounds, the lowest IC50 and Ki values of KFP_H008 consistent with the prediction results that KFP_H008 presented the longest pH > 4 holding time and a relatively short onset time at various doses. It is also worth noting that, KFP_H008 didn’t exhibit the most favourable PK properties in the simulations. In Figure 5A, compound-2 demonstrated a rapid attainment of peak plasma concentration following oral administration. Leveraging the AI-PBPK platform can aid in the identification of the optimal combination of PK, PD and doses of the high-quality drug candidates throughout the compound screening process. Among the five compounds, KFP_H008 presented the best therapeutic effects.

In summary, the platform can leverage a combination of machine learning and PBPK model to predict drug efficacy during the discovery stage. It establishes a direct link between clinical endpoints and structures that influence PK and/or PD outcomes. One of the benefits of employing AI in predicting drug parameters for PBPK models is the capability to analyse multiple drug classes, not just a single category. Currently the training database in the B^2^O platform included thousands of training data which covered most of the main chemical structures in drugs. Provided that appropriate calibration and validation steps are performed prior to prediction, the platform should be capable of reliable predicting PK and PD outcomes within the same category to find out the best ‘me too’ drugs. In the future, when the first-in-class drug database is created, virtual drug screening will be available to predict PK and/or PD outcomes for first-in-class drugs.

## 5 Conclusion

As a simulation tool, the AI-PBPK/PD platform showed the potential to predict the desired therapeutic effects of drug candidates at the early drug discovery stage. Among the five compounds, KFP_H008 presented the best therapeutic effects, with the longest pH > 4 holding time and a relatively short onset time when administered at doses exceeding 10 mg.

## Data Availability

The original contributions presented in the study are included in the article/Supplementary Material, further inquiries can be directed to the corresponding authors.
